# Novel Speech Recognition Systems Applied to Forensics within Child Exploitation: Wav2vec2.0 vs. Whisper

**DOI:** 10.3390/s23041843

**Published:** 2023-02-07

**Authors:** Juan Camilo Vásquez-Correa, Aitor Álvarez Muniain

**Affiliations:** Fundación Vicomtech, Basque Research and Technology Alliance (BRTA), Mikeletegi 57, 20009 Donostia-San Sebastián, Spain

**Keywords:** speech recognition, keyword spotting, child abuse, federated learning, Whisper, Wav2vec2.0

## Abstract

The growth in online child exploitation material is a significant challenge for European Law Enforcement Agencies (LEAs). One of the most important sources of such online information corresponds to audio material that needs to be analyzed to find evidence in a timely and practical manner. That is why LEAs require a next-generation AI-powered platform to process audio data from online sources. We propose the use of speech recognition and keyword spotting to transcribe audiovisual data and to detect the presence of keywords related to child abuse. The considered models are based on two of the most accurate neural-based architectures to date: Wav2vec2.0 and Whisper. The systems were tested under an extensive set of scenarios in different languages. Additionally, keeping in mind that obtaining data from LEAs are very sensitive, we explore the use of federated learning to provide more robust systems for the addressed application, while maintaining the privacy of the data from LEAs. The considered models achieved a word error rate between 11% and 25%, depending on the language. In addition, the systems are able to recognize a set of spotted words with true-positive rates between 82% and 98%, depending on the language. Finally, federated learning strategies show that they can maintain and even improve the performance of the systems when compared to centralized trained models. The proposed systems set the basis for an AI-powered platform for automatic analysis of audio in the context of forensic applications of child abuse. The use of federated learning is also promising for the addressed scenario, where data privacy is an important issue to be managed.

## 1. Introduction

The growth in online child exploitation and abuse material is a significant challenge for European Law Enforcement Agencies (LEAs). Currently, the revision of online material about child abuse exceeds the capacity of LEAs to respond in a practical and timely manner. One of the most important sources of information that needs to be analyzed to find evidence about child abuse corresponds to audiovisual material from multimedia content. With the aims of safeguarding victims, prosecuting offenders and limiting the spread of online child abuse related material, LEAs need a next-generation AI-powered platform to process multimedia data from online sources. One of the main goals of the GRACE project (https://www.grace-fct.eu/ accessed on 1 February 2023) is to develop robust AI-based technology to equip LEAs with the aforementioned platform. Two of the core applications to be incorporated in order to accurately transcribe audiovisual online material and to detect the presence of specific keywords about child abuse in the transcriptions are automatic speech recognition (ASR) and keyword spotting (KWS).

Within this context, ASR technology has been applied in various forensic scenarios—for instance, to collect evidence via the examination of electronic devices [1] or to analyze multimedia content related to specific threats [2,3]. Nevertheless, the successful implementation of an ASR system in forensics introduces a series of issues to be solved, which are not present in other domains where ASR is applied. For instance, it is common to find audio coming from different sources, which are highly affected by background noise, overlapping speakers, and audio reverberation, among other factors. All these aspects affect the quality of the obtained transcription and the capability of the system to detect specific keywords.

Despite the aforementioned problems, recent advances in ASR have introduced novel end-to-end architectures [4] that have shown to be accurate enough in those adverse conditions. The core idea of end-to-end models is to directly map the input speech signal to character sequences and therefore greatly simplify training, fine-tuning, and inference making [5,6,7,8,9]. Two main approaches are distinguished in the literature to train end-to-end ASR systems: fully supervised or self-supervised models. Regarding the first group, NVIDIA proposed Quartznet [10] with the aim of building a competitive but lighter end-to-end ASR model. The architecture consists of multiple blocks of 1D convolutions stacked with residual connections. The model has been trained and tested on the Common Voice corpus, achieving word error rates (WERs) between 7.7% and 12.5%, depending on the language [11]. A Quartznet model also produced WERs of 19.2% and 18.3% in French and Spanish language multimedia data, respectively, from the MediaSpeech corpus [12]. Researchers from NVIDIA recently proposed Citrinet [13] as an evolution of Quartznet. The model consists of a residual network formed by 1D time-channel separable convolutions combined with a sub-word encoding and a squeeze-and-excitation mechanism [14]. The authors reported a WER of 5.6% on the TEDLIUMv2 corpus. Another architecture that has proven to be accurate in many ASR benchmark scenarios is the recurrent neural network transducer (RNN-T) [15]. The RNN-T is formed by three main blocks: (1) an encoder network that receives input acoustic frames and produces high-level speech representations, (2) a predictor that acts as a decoder by processing the previous produced token, and (3) a joint network that combines the outputs from the two previous blocks and produces the distribution of the next predicted token or blank symbol. Recent models based on RNN-T achieved a WER 14.0% in the TEDLIUMv2 corpus [16].

Contrary to fully supervised models, recent studies are focused on the use of big acoustic models trained with self-supervised learning methods and a large amount of unlabeled data. Researchers from Meta AI demonstrated the capabilities of models of this type by introducing Wav2Vec2.0 [17]. This system outperformed many benchmark results, especially when considering ASR for low-resource languages in the Common Voice corpus [18]. Particularly, the authors in [19] considered a Wav2vec2.0 model combined with their proposed language modeling approach and achieved state-of-the-art results in the German Common Voice corpus, with a WER of 3.7%. Wav2Vec2.0-based models have also been successfully tested in more adverse acoustic environments, such as in multimedia Portuguese data from the CORAA database [20]. Due to these reasons, Wav2Vec2.0 has become one of the most often considered neural-based models for ASR. Self-supervised approaches such as Wav2Vec2.0 are challenging because there is not a predefined lexicon for the input sound units during the pre-training phase. Moreover, sound units have variable length with no explicit segmentation [21]. With the aim of solving such issues, Meta AI released HuBERT as a new approach to learn self-supervised speech representations [22]. The combination of convolutional and transfomer networks from Wav2Vec2.0 and HuBERT has achieved state-of-the-art results in many ASR scenarios. With the aim of combining the best features from both type of networks in a single neural block, researchers from Google introduced the “convolutional augmented transfomer” or Conformer [23]. A Conformer network achieved a WER of 7.2% in the TEDLIUMv2 corpus [24].

Self-supervised audio encoders like Wav2Vec2.0, HuBERT, and Conformers learn high quality audio representations. However, due to its unsupervised pre-training nature, they lack a proper decoding to transform such representations into usable outputs. This is why a fine-tuning stage is always necessary in order to accurately implement models for ASR or audio classification. With the aim of solving the aforementioned issue, researchers from OpenAI recently proposed “Whisper” [25]. Whisper is a sequence-to-sequence transformer trained in a fully supervised manner, using up to 680,000 h of labeled audio from the Internet. The model has achieved state-of-the-art WER results on many benchmark datasets for ASR, including librispeech, TEDLIUM, and Common Voice, among others.

There are two main issues that appear when designing ASR solutions for forensic scenarios: The first one is related to find the most appropriate neural architecture from the ones previously described in order to deal with different acoustic environments. The second one is related to data privacy and protection [26]. Generally, obtaining operative data from LEAs for the addressed scenario is not possible. In this context, federated learning (FL) has emerged as an alternative with which to train machine learning models on remote devices, such as mobile phones and remote data-centers in a non-centralized manner, preserving data privacy [27,28,29,30]. The procedure is as follows: LEAs’ operative data are stored in on-premise data servers. Then, FL strategies aim to transfer only local model updates to a central server, keeping LEAs’ data private. The central server aggregates information obtained from multiple clients, i.e., LEAs, and updates a central model that is transmitted back to the clients for their consumption. FL has been applied to train robust federated acoustic models for ASR [31,32,33] and KWS [34]. In [32], the authors proposed a client-adaptive federated training scheme to mitigate data heterogeneity when training ASR models. The proposed system achieved a similar WER with respect to the obtained one using fully centralized training. In [33], the authors proposed a strategy to compensate non-independent and identically distributed (non-IID) data in federated training of ASR systems. The proposed strategy involved random client data sampling, which resulted in a cost-quality trade-off. The optimization of such a trade-off led to obtaining ASRs with similar WERs to the obtained by training-centralized systems. The authors in [34] demonstrated the capabilities of federated training to obtain robust KWS systems locally trained on edge devices such as smartphones, reaching similar accuracies when compared with centralized trained models.

According to the reviewed literature, the two main paradigms and solutions for ASR to date include self-supervised models based on Wav2Vec2.0 and fully supervised models such as Whisper. This work considered and compared these two approaches to test their capabilities to perform robust ASR and KWS in a large set of test scenarios. We also evaluated the use of FL in the context where different LEAs can share a common ASR and KWS system, keeping the privacy of their data. In summary, the main contributions of this paper are four-fold:

1. We performed an extensive comparison between two of the most accurate neural-based ASR architectures to date: a fine-tuned version of Wav2Vec2.0 and Whisper. The evaluation was performed in many scenarios, but paying special attention to corpora coming from multimedia content. The models were tested on data from seven indo-European Languages, including English, Spanish, German, French, Italian, Portuguese, and Polish. This evaluation can be useful in other domains besides ASR forensics, making our contribution open and viable for other scenarios.
2. We created and released an in domain corpus that includes specific keywords of the child abuse domain, and a set of accompanying audio files where the keywords are present. The included audio was selected from open available corpora used in the literature. The created corpus can be used as a benchmark to test ASRs in uncontrolled acoustic conditions.
3. The two neural architectures are compared as well in the created corpora within the scope of child abuse forensics. To the best of our knowledge, this is the first study to comprise the use of open ASR solutions and their capabilities to recognize specific words within a forensic domain.
4. We validated the use of FL strategies to train ASR systems in the context of forensic applications. The core idea is that different LEAs can share a common model while keeping the privacy of their data.

The rest of the paper is distributed as follows. Section 2 details different technical aspects of Wav2Vec2.0 and Whisper architectures for ASR. Section 3 describes the considered corpora to test the ASR systems, and the process to deliver an in domain corpus for KWS in the context of forensics. Section 4 describes the pilot study on the use of FL for the addressed application. Section 5 displays the main results obtained regarding ASR, KWS, and FL. Section 6 discusses the main insights obtained from the results. Finally, Section 7 shows the main conclusion derived from this work.

## 2. Methods

We considered two of the most accurate neural-based ASR architectures to date: (1) Wav2vec2.0, which is trained following a self-supervised paradigm, and (2) Whisper, which is trained following a fully supervised strategy. Details about each model are found in the following sub-sections.

### 2.1. Wav2vec2.0

**Figure 1:**
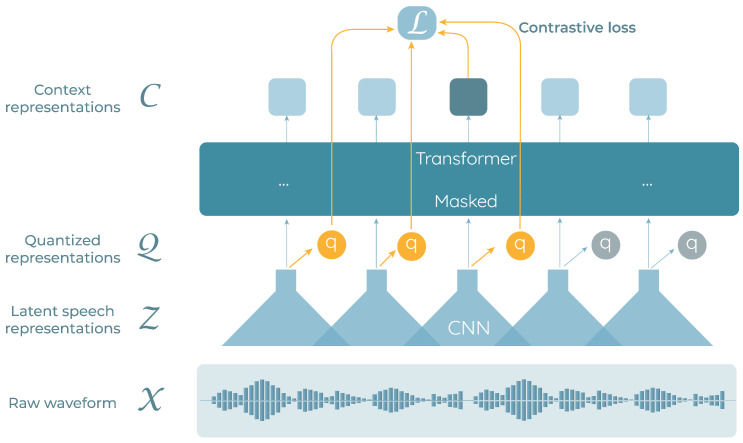
Wav2vec2.0 architecture representation. The raw audio signal is mapped to speech representations that are fed into a transformer network to output context representations. Figure based on the one presented in [17].

Wav2vec2.0 [17] is a self-supervised end-to-end architecture based on convolutional and transformer layers (see Figure 1). The model encodes raw audio waveforms χ into latent speech representations $z_{1},\ldots ,z_{T}$ via a multi-layer convolutional feature encoder f:χ→Z. These latent representations fed a transformer-masked network g:Z→C. The transformer network initially quantizes the continuous representations, forming a discrete set of outputs $q_{1},\ldots ,q_{T}$ that represent targets in the self-supervised learning objective [17,35]. Those quantized representations are then contextualized using the attention blocks from the transformer module, obtaining a set of discrete contextual representations $c_{1},\ldots ,c_{T}$. The feature encoder is formed by seven convolutional blocks with 512 channels, strides of {5,2,2,2,2,2,2} and kernel widths of {10,3,3,3,3,2,2}. The transformer network is formed by 24 blocks, 1024 dimensions, inner dimensions numbering 4096, and a total of 16 attention heads.

We considered a pre-trained Wav2vec2.0 acoustic model based on the Wav2Vec2-XLS-R-300M model, which is available via Hugginface (https://huggingface.co/facebook/wav2vec2-xls-r-300m accessed on 1 February 2023). The model was pre-trained in a self-supervised manner using 436k hours of unlabeled speech data in 128 languages from the VoxPopuli [36], Multilingual librispeech (MLS) [37], Common Voice [38], BABEL, and VoxLingua107 [39] corpora. The Wav2Vec2-XLS-R-300M is one of the different versions of the Meta AI’s XLS-R multilingual model [40] composed by 300 million parameters. The multilingual pre-trained model was fine-tuned with labeled speech data (see Section 3.1) in seven languages: English, German, French, Spanish, Italian, Portuguese, and Polish. Each model was trained for 50 epochs, with a batch size of 2, 16 gradient accumulation steps, and a learning rate of $5\times 10^{-5}$, which was warmed up during the initial 10% of the training.

The trained acoustic representations were decoded using a connectionist temporal classification (CTC) layer with a beam-search decoding strategy (beam-width = 256). The CTC decoding included the use of separate 3-gram language models that are trained using large text corpora, and which were included in the decoding with weights of α=0.5 and β=1.5.

### 2.2. Whisper

**Figure 2:**
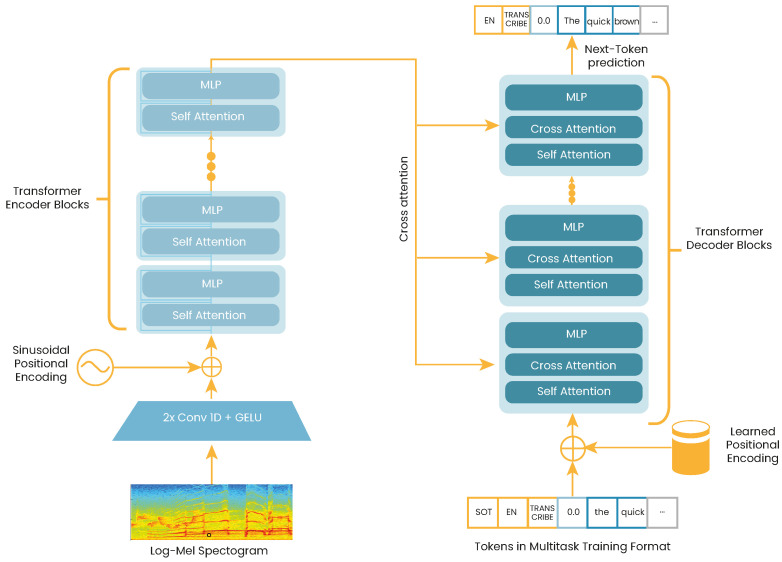
Whisper architecture representation. The log Mel-spectrograms are encoded by a transformer network. Encoded representations are transformed into character outputs and no-speech tokens via the transformer decoder. Figure based on the one presented in [25].

Whisper is a recently introduced ASR system by OpenAI [25]. Contrary to Wav2vec2.0, Whisper is trained in a fully supervised manner, using up to 680k hours of labeled speech data from multiple sources. The model is based on an encoder-decoder Transformer, which is fed by 80-channel log-Mel spectrograms. The encoder is formed by two convolution layers with a kernel size of 3, followed by a sinusoidal positional encoding, and a stacked set of Transformer blocks. The decoder uses the learned positional embeddings and the same number of Transformer blocks from the encoder. Figure 2 illustrates the general Whisper architecture. Different pre-trained models are available with variations in the number of layers and attention heads. We considered the "Whisper-large" model, which consists of 1550 million parameter distributed in 32 layers and 20 attention heads. The model is available via Huggingface (https://huggingface.co/openai/whisper-large accessed on 1 February 2023).

The model was not fine-tuned in this study; thus, the evaluation for all languages was conducted in a zero-shot setting. The decoding was performed using a beam search strategy with 5 beams, an array of temperature weights of $\left[0.2,0.4,0.6,0.8,1\right]$, and a no repeat *n*-gram size of 3 in order to take advantage of the language modeling head and to avoid loops, in a similar way to [25].

## 3. Materials

This section describes a set of open corpora used to benchmark the two considered ASR systems (Section 3.1), followed by the process performed to derive a set of keywords to be spotted by the considered systems (Section 3.2), and the description of a built in domain dataset considered as well to test the considered models (Section 3.3).

### 3.1. Data

The ASR and KWS models were trained an evaluated in a set of seven indo-European languages: English, Spanish, German, French, Italian, Portuguese, and Polish. These languages were selected because of two main reasons: (1) we covered German, Latin, and Slavik-based languages, which represent the majority of type of languages spoken in Europe, and (2) these languages were particularly selected by the Law Enforcement Agencies (LEAs) for the applications related to detecting child-abuse in online sources. Different public corpora were considered to train/test the ASR and KWS models in each language. Wav2vec2.0 models were fine-tuned using the Common Voice corpus [38] for each considered language. The amount of available labeled data highly varies depending on the language, and include: 1600 h for English, 777 h for German, 623 for French, 324 for Spanish, 158 for Italian, 63 for Portuguese, and 43 for Polish. These data are freely available via Huggingface (https://huggingface.co/datasets/common_voice accessed on 1 February 2023). The training data for the Spanish model also included 57 h from the RTVE2018 dataset [41] from the Albayzin 2018 evaluation challenge.

The corpora covered in our paper include both European and American accents for the aforementioned languages. In addition, the common voice corpus, which was used as our train set, was crowd-sourced from many countries and includes a large number of accents that helps to improve the generalization capabilities of our models.

The performance of the fine-tuned Wav2vec2.0 and the Whisper-based models were evaluated in a cross-corpora fashion, considering a large set of databases from the literature that are available in the different languages. The list of considered corpora is observed in Table 1. These corpora were selected in order to test the performances of the models in several recording conditions, which can be more similar to the realistic scenarios found by LEAs. Notice that due to the sensitive nature of the target application, it is not possible to get access to realistic operative data from LEAs. However, we created an in-domain synthetic dataset using these open source corpora, which is described in Section 3.3.

### 3.2. Spotted Keywords

**Figure 3:**
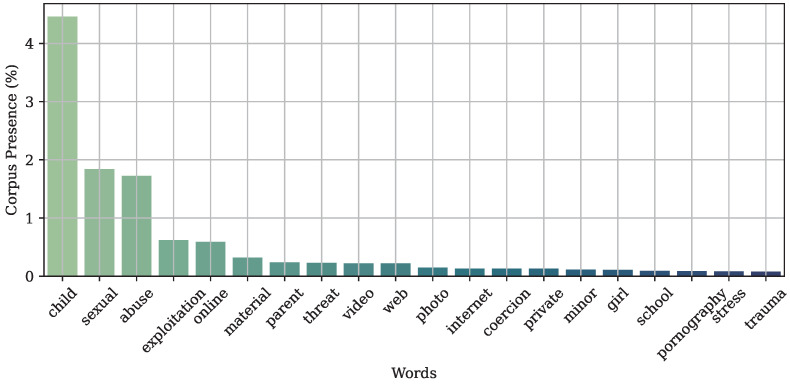
Top 20 of the most important keywords related to child abuse, which were used to test the capability of the ASR system to detect specific terminology within the domain.

In order to test the capabilities of the ASR models to spot specific keywords within the child abuse domain, we defined a list of keywords to be spotted. The keyword list was obtained from a set of open documents that include: (1) the “Best Practices on Victim support for LEA first responders” deliverable from the GRACE project (https://www.grace-fct.eu/deliverables/70 accessed on 1 February 2023), (2) the 2021 “Barriers to Compensation for Child Victims of Sexual Exploitation” report from ECPAT (https://ecpat.org/wp-content/uploads/2021/05/Barriers-to-Compensation-for-Child_ebook.pdf accessed on 1 February 2023) [47], (3) the study from [48], (4) EUROPOL technical reports [49,50,51], (5) EUROPOL press-releases from 2018 to 2022 using the keyword “child abuse” (https://www.europol.europa.eu/media-press/newsroom?q=child%20abuse accessed on 1 February 2023), (6) Wikipedia articles about “child abuse” and “online child abuse”, and (7) UNICEF press-releases about “child abuse” (https://www.unicef.org/search?force=0&query=child+abuse&created%5Bmin%5D=&created%5Bmax%5D= accessed on 1 February 2023). All documents were text crawled and pre-processed by performing lemmatization and removing stop words, numbers, and date entities. After this process, we obtained a corpus with 55,059 words, of which 6028 are unique. Figure 3 shows the most important keywords found in the crawled corpus.

Afterwards, we selected the 100 most repeated words from the corpus, which represent 33% of the information within the whole set of crawled documents. Finally, we excluded 12 terms because they were very broad concepts not related to child abuse, leading to a final set of 88 keywords to be spotted. The obtained keyword list (in English) was translated into the remaining six considered languages in order to have a common benchmark for all languages.

### 3.3. GRACE Dataset

We considered an additional corpus to test the implemented ASR systems by merging and filtering the data described in Section 3.1. We selected audio samples from all datasets that contain at least one of the 88 selected keywords. Table 2 shows the data distribution for each language after selection. The table includes the datasets considered for each language where the keywords were found, the number of utterances, and the total audio duration (in hours).

The selected audio files were processed in order to have more realistic acoustic conditions, like those expected in forensic applications within the considered domain. The process included: (1) adding background noise with signal to noise ratios (SNR) between 5 and 30 dB (randomly), (2) adding reverberation using room impulses from the VOiCES dataset [52], and (3) randomly applying the ogg-vorbis codec [53] due to it being commonly found in audio material from online sources. The final ASR and KWS evaluation was performed considering the two versions of the corpus: clean and noisy. This corpus is available online (https://datasets.vicomtech.org/di01-grace-automatic-speech-recognition-and-keyword-spotting/GRACE_ASR.zip accessed on 1 February 2023) to be used as a benchmark dataset for speech recognition in different languages under uncontrolled acoustic conditions.

## 4. Federated Learning

**Figure 4:**
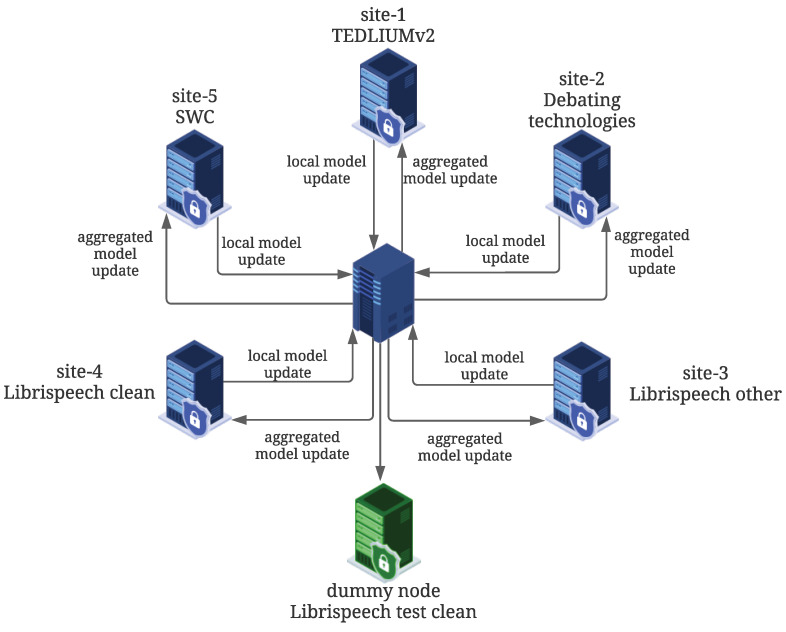
Configuration of the FL architecture. Central server with five client nodes (site-$\left\{1,2,\ldots ,5\right\}$) and a dummy node only used to test the performance of the aggregated model.

The considered FL pipeline was performed only with English data and included five nodes that were used for federated training, a dummy node considered to test the evolution of the learning process, and a central server in charge of aggregating the weights received from the five nodes. Figure 4 shows the implemented architecture. Three of the servers were located at Vicomtech premises (Spain), one server was located in Greece, another one in Portugal, and the remaining one in Cyprus. The aim of these connections was to create a real environment for the pilot, in similar conditions to the expected one when the model will perhaps be trained by different LEAs across Europe. In addition, secure communication between clients and the server was established through a VPN connection to ensure that sensitive data (parameters) were safely transmitted and to prevent unauthorized access. Each node contained data from a different dataset: TEDLIUMv2, debating technologies, Librispeech-other, Librispeech-clean, and SWC. This data configuration aimed to evaluate the impact of non-IID data distribution, which is more realistic for the addressed forensic application.

The FL pilot test was performed only with the Wav2Vec2.0 model and with the pre-trained Wav2Vec2-XLS-R-300M model. The training hyperparameters were the same for the five clients, and included a batch size of 2, a learning rate of $5\times 10^{-5}$ warmed up in the first 10% of the training time, and a gradient accumulation of 16 steps. The local training was performed for 5 epochs. The central server was configured to run for 10 rounds of federated training, while using the federated averaging (FedAvg) aggregation mechanism to update the central model. The architecture configuration and the training process were implemented using Nvidia Flare (https://nvflare.readthedocs.io/en/main/index.html accessed on 1 February 2023).

## 5. Results

### 5.1. Speech Recognition

Wav2Vec2.0 and Whisper models were evaluated under the corpora described in Section 3.1. The results of the ASR systems in terms of WER are shown in Table 3. The results included those obtained in the evaluation of the seven languages, and using both the open benchmark corpora and the two versions (clean and noisy) of the synthetic GRACE corpus.

**Figure 5:**
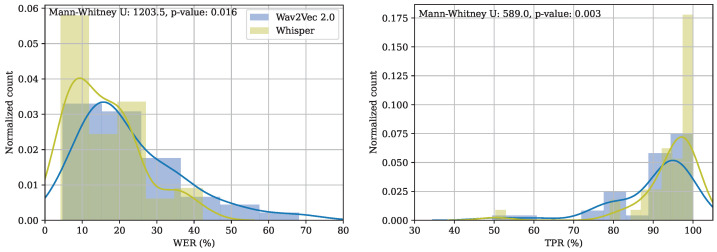
Comparison between the results obtained using Wav2Vec2.0 and Whisper for ASR (**left**) and KWS (**right**).

On average, the WER for each language using Whisper ranged from 11.3% (in Spanish) to 24.9% (in French). The results using Wav2Vec2.0 ranged from 13.1% (in Spanish) to 34.8% (in Portuguese). In general, Whisper produces less errors than Wav2Vec2.0 (see Figure 5 left). The difference between the models was statistically significant according to a Mann–Whitney test (U = 1203.5, *p*-value = 0.016). Whisper outperformed Wav2Vec2.0, especially under the most affected acoustic conditions, such as in the GRACE noisy, TEDLIUMv2, Debates, and CORAA corpora. However, there are some scenarios where Wav2Vec2.0 outperformed Whisper and which should be considered with special attention, such as the results for Spanish Common Voice.

The results obtained were compared to those found in the literature for the multilingual corpora: Common Voice, MLS, MTEDx, and MediaSpeech. The comparison is shown in Table 4. The Wav2Vec2.0-based model outperformed results in the Spanish versions of Common Voice and MediaSpeech corpora, with WERs of 4.3% and 14.5%, respectively, with respect to the results reported in [18] for Common Voice (WER = 6.2%) and in [12] for MediaSpeech (WER = 18.3%). We also reported state-of-the-art results for the Spanish, Portuguese, Italian, and German versions of the MTEDx corpus (WERs of 9.4%, 12%, 11.6%, and 21.7%, respectively) with respect to the WERs of 16.2%, 20.2%, 16.4%, and 42.3% reported in [43]. The Whisper model also achieved state-of-the-art results on the CORAA corpus (WER = 21.7%) with respect to the results reported in [54] (WER = 21.9%), and on the TEDLIUMv2 corpus (WER = 5.4%) compared to [13] (WER = 5.6%). Regarding MLS, the state-of-the-art results are still from [55]. However, notice that the results reported here correspond to cross-corpus tests, whereas the experiments performed in [55] were on Wav2Vec2.0 models trained and tested using MLS, thereby making the models adapted just for such a corpus.

### 5.2. Keyword Spotting

The text transcriptions from Wav2Vec2.0 and Whisper were post-processed in order to find the presence of the defined keywords to be spotted. The process involved transforming the transcription to lowercase and lemmatization. Lemmatization was performed to reduce the inflectional form of each word in order to detect all possible variations of the word within the transcription. The lemmatization process was performed using the set of large open dictionaries available in Spacy (https://spacy.io/usage/models accessed on 1 February 2023). The results obtained for KWS in each corpus are shown in Table 5. The results are presented in terms of the true positive rate (TPR). This is a common metric used in applications of this type where it is more important to avoid false-positive than false-negative errors [58,59].

On average, the TPRs were higher using Whisper, and the results per language using Whisper ranged from 81.5% (for Polish) to 98.4% (for Italian). Results using Wav2Vec2.0 ranged from 82.9% (for Portuguese) to 94.9% (for Spanish). Similarly to the ASR results, the difference between Whisper and Wav2Vec2.0 was larger when considering speech signals in uncontrolled acoustic conditions, such as the ones from the GRACE noisy corpus, where we can guarantee the presence of the spotted keywords in every utterance. High differences were also observed for the CORAA corpus, Common Voice, and the German SWC. The differences between the results obtained using Wav2Vec2.0 and Whisper were also statistically significant (see Figure 5 right) according to a Mann–Whitney test with U = 589.0 and a *p*-value = 0.003.

### 5.3. Federated Learning

The FL experiment involved training the Wav2Vec2.0 system using five separate real servers for training, and one additional node (dummy) used only to test the final model. Each node contained data from a different dataset (only in English) in order to evaluate the contribution from each corpus to the global aggregated model. The aim was also to cover non-IID conditions, which have shown to be one of the most important drawbacks when training models in an FL approach. The results are shown in Table 6. The results using the FL training are compared to those obtained when training the system in a completely centralized manner. Similar WERs were obtained by each node in the federated and centralized training. The main difference is that when considering FL models, there is only one aggregated model which covers the results of the five nodes, instead of having five different models for the case of the centralized approach. This fact greatly reduces the time considered to train the system, and most importantly, it is possible to take advantage of data from different data centers to train a more robust and general model without the need for sharing data among clients.

## 6. Discussion

The evaluation of Wav2Vec2.0 and Whisper-based ASR systems was performed in a large set of different scenarios, including one specifically designed for forensic applications within the child abuse domain. On average, Whisper is more accurate than the Wav2Vec2.0-based system. Whisper achieved WERs ranging from 11.5% to 24.9%, depending on the language, compared with Wav2Vec2.0’s WERs of between 13.3% and 34.8%. The difference between the two models was even larger when using languages trained with fewer resources, such as Portuguese or Italian. Despite these differences, Wav2vec2.0 is competitive with Whisper when the number of hours for fine-tuning is large, e.g, for English, Spanish, or French.

Results using the GRACE dataset showed relatively similar WERs between Wav2Vec2.0 and Whisper when considering the clean version of the corpus: the average WER was 22.1% for Whisper and was 23.2% for Wav2Vec2.0. However, the difference between the two models greatly increased with the noisy version of the corpus: the average WER was 26.3% for Whisper and 45.1% for Wav2Vec2.0. This is a great indicator of the ability of Whisper to perform accurate transcriptions under uncontrolled and noisy acoustic conditions, by keeping similar WERs in the two versions of the GRACE corpus. Despite the differences between the two types of models, there are some surprising results where Wav2Vec2.0 outperformed Whisper, and which should be considered with special attention—for instance, when evaluating the GRACE clean corpus in languages such as English, French, and Spanish. The models for these three languages were fine-tuned with more data, which likely explains the lower WER for Wav2Vec2.0 compared to that of Whisper.

Our systems achieved state-of-the art results on several of the considered benchmark corpora. We reported state-of-the-art results for some of the languages in the Common Voice corpus. State-of-the-art results were also achieved for almost all languages ib the MTEDx and MediaSpeech corpora. These results are good indicators if the ability of the considered systems to accurately recognize speech under more natural and spontaneous scenarios, closer to the expected in forensic domains.

The KWS evaluation indicated that both Wav2Vec2.0 and Whisper were accurate enough to recognize the considered child-abuse-related keywords in the seven languages. TPRs obtained for Wav2Vec2.0 ranged from 82.9% to 94.9%, depending on the language. Results using Whisper ranged from 80.3% to 98.2%. The particular evaluation of KWS in the GRACE dataset also showed that both models are equally accurate at recognizing the selected keywords under controlled acoustic conditions. On the contrary, when considering the noisy version of the corpus, the results for Wav2Vec2.0 were reduced by 20%, and the results for Whisper were only reduced by 3%. This fact again indicates the ability of Whisper to accurately process speech recordings in uncontrolled acoustic conditions.

The last experiment involved a pilot study on the use of FL to train ASR systems. The results indicated that an ASR trained in a federated way maintains and in some cases outperforms the performance of individual ASRs trained in a centralized manner by each LEA. In addition to the performance, the most important aspect of FL is that the ASR training does not involve any data sharing among LEAs, since only updates of the network parameters are transferred to a central server in charge of aggregating the model. These results are indicators of the potential use of FL to obtain a joint (and potentially richer) model combining sources of data that could not be otherwise combined. Despite the benefits of using FL, it is important to consider external factors that may degrade the performance and reliability of the system. For instance, there is evidence of FL attacks that are able to retrieve speaker information from the transferred weights [60] and data poisoning attacks inside LEA servers. Different strategies can be considered to mitigate attacks of these kinds, such as the use of differential privacy algorithms [61] or the use of trusted execution environments.

## 7. Conclusions

This paper proposed the use of speech recognition and keyword spotting technologies to be applied in forensic scenarios, particularly in child exploitation settings. The aim is to provide LEAs with technology to detect the presence of offensive online audiovisual material related to child abuse. State-of-the art ASR systems based on Wav2Vec2.0 and Whisper were considered for the addressed application. The performance of both models was tested on a large set of open benchmark corpora from the literature. Therefore, the results obtained can be extended to other ASR domains. We additionally created an in-domain corpus using different open source datasets from the research community. The aim was to test the models in more realistic and operative conditions.

The ASR and KWS models were evaluated in corpora from seven Indo-European languages, including English, German, French, Spanish, Italian, Portuguese, and Polish. We obtained overall WERs ranging from 11.3% to 24.9%, depending on the language. The performance of the KWS model for the different languages ranged from 81.5% to 98.4%. The most accurate results were obtained from models trained with more data, such as English or German. The comparison between Wav2Vec2.0 and Whisper models indicated that the second one was the most accurate system in the majority of cases, especially when considering utterances in uncontrolled acoustic conditions.

We also proposed a strategy for using FL to train robust ASR systems in the context of the addressed application. This is a suitable approach considering that collecting operational data from LEAs is not possible. FL approaches allow LEAs to build a common technological platform without the need to share their operational data. The results of the FL pilot indicated that similar WERs were achieved when comparing the model trained in a federated way to individual models trained in a centralized manner, even considering non-IID conditions, which has been shown to be one of the main drawbacks in FL.

For future work, the considered approaches can be extended to other forensic applications where there is a need to monitor audiovisual material from online sources. In addition, the considered technology can be combined with other speech processing methods, such as speaker and language identification, age and gender recognition, and speaker diarization. The ultimate goal is to provide LEAs with accurate tools to monitor audio from online sources, allowing them to respond in a practical and timely manner.

## Figures and Tables

**Table 1:** List of public speech corpora considered to test the performances of ASR and KWS systems based on Wav2Vec2.0 and Whisper.

Corpus Name	Description	Languages	Test Duration (h)
Common Voice [38]	Read sentences collected and validated via crowd-sourcing	English	173
		German	72
		French	38
		Spanish	26
		Italian	23
		Portuguese	6
		Polish	7
Spoken Wikipedia Corpus (SWC) [42]	Volunteer readers of Wikipedia articles	English	42
		German	36
Media Speech [12]	Speech segments from YouTube videos	French	10
		Spanish	10
Multilingual TEDx [43]	Audio recordings and transcripts from TED talks	German	2
		French	2
		Spanish	2
		Italian	2
		Portuguese	2
TEDLIUMv2 [44]	Audio recordings from TED talks	English	3
Multilingual librispeech (MLS) [37]	Audio recordings from audiobooks	German	14
		French	10
		Spanish	10
		Italian	5
		Polish	2
		Portuguese	4
Voxforge	Crowdsourced read speech	German	3
		French	4
		Spanish	5
		Italian	2
		Portuguese	1
Debating technologies [45]	Audio recordings from transcribed public debates	English	1
Polish Parliamentary corpus [46]	Recordings from the Polish parliament	Polish	1
CORAA [20]	Combination of five corpora in Portuguese	Portuguese	13

**Table 2:** Data distribution for the GRACE dataset, which combines different corpora into a single one within the child abuse domain.

Language	Base Corpora	# Utterances	Duration (h)
English	SWC, Debating technologies, TEDLIUMv2	2979	9.2
German	Multilingual TEDx, SWC, Voxforge	1712	5.9
French	Multilingual TEDx, MediaSpeech, Voxforge	1250	4.1
Spanish	Multilingual TEDx, MediaSpeech, Voxforge	557	2.0
Italian	Multilingual TEDx, Voxforge	354	1.0
Portuguese	Multilingual TEDx, Voxforge, CORAA	1503	2.3

**Table 3:** Results of the ASR models in different languages considering all benchmark datasets. Results in terms of WER.

Model	Common	MLS	TED-	MTEDx	SWC	Media	Voxforge	Debates	Polish	CORAA	GRACE	GRACE	AVG.
	Voice		LIUMv2			Speech			Parl		Clean	Noisy	
English													
Wav2Vec 2.0	16.1	-	17.2	-	20.6	-	-	11.7	-	-	18.9	32.6	19.5
Whisper	10.0	-	5.4	-	20.6	-	-	7.0	-	-	24.5	19.8	14.6
German													
Wav2Vec 2.0	11.9	12.9	-	36.7	34.5	-	7.5	-	-	-	20.0	33.8	22.5
Whisper	7.1	6.7	-	21.7	18.3		4.2	-	-	-	15.5	22.5	13.7
French													
Wav2Vec 2.0	16.7	17.0	-	25.3	-	29.1	16.7	-	-	-	26.5	56.3	26.8
Whisper	21.7	8.0	-	23.3	-	35.8	14.6	-	-	-	36.8	34.1	24.9
Spanish													
Wav2Vec 2.0	4.7	7.2	-	12.9	-	14.5	6.3	-	-	-	12.6	33.3	13.1
Whisper	6.2	5.3	-	9.4	-	15.8	4.2	-	-	-	19.6	18.8	11.3
Italian													
Wav2Vec 2.0	12.8	21.1	-	22.2	-	-	14.3	-	-	-	18.0	46.3	22.5
Whisper	7.9	13.6	-	11.6	-	-	10.5	-	-	-	14.1	20.2	13.0
Portuguese													
Wav2Vec 2.0	12.9	20.1	-	33.8	-	-	17.8	-	-	48.5	42.7	68.1	34.8
Whisper	5.4	8.8	-	13.1	-	-	11.2	-	-	21.7	22.1	42.3	17.8
Polish													
Wav2Vec 2.0	11.5	12.7	-	-	-	-	-	-	32.1	-	-	-	18.8
Whisper	8.9	6.0	-	-	-	-	-	-	32.5	-	-	-	15.8

**Table 4:** WER comparison between the results reported and those from the state-of-the-art for Common Voice, MLS, and MTEDx corpora. The best result for each corpus and language is highlighted in bold.

Corpus	Reference	Language						
		English	German	French	Spanish	Italian	Portuguese	Polish
Common Voice	[11]	-	7.7	12.5	10.9	-	-	-
	[18]	-	7.2	11.2	6.2	**6.5**	6.1	**7.6**
	[36]	-	7.8	**9.6**	10.0	-	-	-
	[25]	10.1	7.7	14.7	6.4	8.1	7.1	9.0
	[19]	-	**3.6**	-	-	-	-	-
	[56]	-	9.8	-	-	-	-	-
	[57]	-	-	-	-	-	9.2	-
	Wav2vec2.0	16.1	11.9	16.7	**4.7**	12.8	12.9	11.5
	Whisper-large	**10.0**	7.1	21.7	6.2	7.9	**5.4**	8.9
MLS	[37]	-	6.5	5.6	6.1	10.5	19.5	20.4
	[40]	-	7.4	10.0	6.9	12.0	15.6	9.8
	[55]	-	**4.1**	**5.0**	**3.7**	**8.2**	**8.0**	**6.6**
	[25]	-	6.6	8.9	5.4	14.3	9.2	**6.6**
	[57]	-	-	-	-	-	12.3	-
	Wav2vec2.0	-	12.9	17.0	7.2	21.1	20.1	12.7
	Whisper-large	-	6.7	8.0	5.3	15.8	8.8	9.9
MTEDx	[43]	-	42.3	**19.4**	16.2	16.4	20.2	-
	[57]	-	-	-	-	-	21.0	-
	Wav2vec2.0	-	36.7	25.3	12.9	22.2	33.8	-
	Whisper-large	-	**21.7**	23.3	**9.4**	**11.6**	**13.1**	-
MediaSpeech	[12]	-	**19.2**	18.3	-	-	-	-
	Wav2vec2.0	-	29.1	**14.5**	-	-	-	-
	Whisper-large	-	35.8	15.8	-	-	-	-

**Table 5:** Results of KWS in different languages considering all benchmark datasets. Results in terms of TPR (%).

Model	Common	MLS	TED-	MTEDx	SWC	Media	Voxforge	Debates	Polish	CORAA	GRACE	GRACE	AVG.
	Voice		LIUMv2			Speech			Parl		Clean	Noisy	
English													
Wav2Vec 2.0	93.3	-	95.4	-	92.5	-	-	96.6	-	-	94.6	79.5	92.0
Whisper	96.8	-	97.4	-	94.1	-	-	97.7	-	-	91.8	93.6	95.2
German													
Wav2Vec 2.0	91.3	96.9	-	93.5	80.4	-	99.8	-	-	-	94.3	79.7	90.8
Whisper	97.8	98.8	-	97.7	97.6		99.8	-	-	-	97.2	90.6	97.1
French													
Wav2Vec 2.0	90.6	90.1	-	**94.5**	-	82.8	90.7	-	-	-	**90.5**	60.1	85.6
Whisper	94.6	98.0	-	93.9	-	84.9	94.2	-	-	-	88.0	81.3	90.7
Spanish													
Wav2Vec 2.0	96.7	98.1	-	98.1	-	96.3	100.0	-	-	-	97.0	78.1	94.9
Whisper	98.2	99.8	-	98.6	-	94.4	99.8	-	-	-	92.0	94.2	96.7
Italian													
Wav2Vec 2.0	90.7	97.2	-	95.5	-	-	98.9	-	-	-	96.1	80.9	93.2
Whisper	97.8	99.9	-	97.3	-	-	99.8	-	-	-	98.7	96.9	98.4
Portuguese													
Wav2Vec 2.0	93.1	93.4	-	94.1	-	-	99.1	-	-	74.3	76.8	49.5	82.9
Whisper	96.6	97.5	-	99.4	-	-	100.0	-	-	88.1	88.3	81.3	93.0
Polish													
Wav2Vec 2.0	93.9	96.9	-	-	-	-	-	-	83.3	-	-	-	91.4
Whisper	95.4	98.7	-	-	-	-	-	-	50.3	-	-	-	81.5

**Table 6:** Results of the FL pilot comparing WERs from Wav2Vec2.0 models trained in a federated or centralized way.

Node	Data	WER Federated	WER Centralized
node-1	TED-LIUMv2	13.5	13.3
node-2	Debates	12.4	12.3
node-3	Librispeech-other	7.9	7.8
node-4	Librispeech-clean	2.8	3.2
node-5	SWC	25.7	24.3
dummy	Librispeech-clean	3.6	3.8

## Data Availability

All data considered in this study are from open repositories under Creative common licenses.

## Abbreviations

ASR	Automatic Speech Recognition
CTC	Connectionist Temporal Classification
FL	Federated Learning
KWS	Keyword Spotting
LEA	Law Enforcement Agency
MLS	Multilingual Librispeech
SWC	Spoken Wikipedia Corpus
TPR	True Positive Rate
